# Flow Cytometry Analysis of Circulating Extracellular Vesicle Subtypes from Fresh Peripheral Blood Samples

**DOI:** 10.3390/ijms22010048

**Published:** 2020-12-23

**Authors:** Marco Marchisio, Pasquale Simeone, Giuseppina Bologna, Eva Ercolino, Laura Pierdomenico, Damiana Pieragostino, Alessia Ventrella, Francesca Antonini, Genny Del Zotto, Daniele Vergara, Christian Celia, Luisa Di Marzio, Piero Del Boccio, Antonella Fontana, Domenico Bosco, Sebastiano Miscia, Paola Lanuti

**Affiliations:** 1 Department of Medicine and Aging Sciences, University “G. d’Annunzio”, Chieti-Pescara, 66100 Chieti, Italy; m.marchisio@unich.it (M.M.); simeone.pasquale@gmail.com (P.S.); giuseppina.bologna@hotmail.it (G.B.); evin30@libero.it (E.E.); laura.pierdomenico@unich.it (L.P.); p.lanuti@unich.it (P.L.); 2 Center for Advanced Studies and Technology (C.A.S.T.), University “G. d’Annunzio”, Chieti-Pescara, 66100 Chieti, Italy; damiana.pieragostino@unich.it (D.P.); piero.delboccio@unich.it (P.D.B.); 3 Department of Innovative Technologies in Medicine & Dentistry, University G. d’Annunzio”, Chieti-Pescara, 66100 Chieti, Italy; 4 Department of Pharmacy, University “G. d’Annunzio”, Chieti-Pescara, 66100 Chieti, Italy; alessia.ventrella@unich.it (A.V.); christian.celia@unich.it (C.C.); luisa.dimarzio@unich.it (L.D.M.); antonella.fontana@unich.it (A.F.); 5 Department of Research and Diagnostics, IRCCS Giannina Gaslini, 16147 Genova, Italy; albarofranci@gmail.com (F.A.); gennydelzotto@gmail.com (G.D.Z.); 6 Laboratory of Clinical Proteomics, “Giovanni Paolo II” Hospital, 73100 ASL-Lecce, Italy; daniele.vergara@unisalento.it; 7 Department of Biological and Environmental Sciences and Technologies, University of Salento, 73100 Lecce, Italy; 8 Department of Biomorphological Science, Molecular Genetic Institute, Italian National Research Council, 66100 Chieti, Italy; domenicoboscotems@yahoo.com

**Keywords:** extracellular vesicles, polychromatic flow cytometry, biomarkers, proteomics, fresh peripheral blood

## Abstract

Extracellular vesicles (EVs) are released by shedding during different physiological processes and are increasingly thought to be new potential biomarkers. However, the impact of pre-analytical processing phases on the final measurement is not predictable and for this reason, the translation of basic research into clinical practice has been precluded. Here we have optimized a simple procedure in combination with polychromatic flow cytometry (PFC), to identify, classify, enumerate, and separate circulating EVs from different cell origins. This protocol takes advantage of a lipophilic cationic dye (LCD) able to probe EVs. Moreover, the application of the newly optimized PFC protocol here described allowed the obtainment of repeatable EVs counts. The translation of this PFC protocol to fluorescence-activated cell sorting allowed us to separate EVs from fresh peripheral blood samples. Sorted EVs preparations resulted particularly suitable for proteomic analyses, which we applied to study their protein cargo. Here we show that LCD staining allowed PFC detection and sorting of EVs from fresh body fluids, avoiding pre-analytical steps of enrichment that could impact final results. Therefore, LCD staining is an essential step towards the assessment of EVs clinical significance.

## 1. Introduction

The information exchange in complex biological systems relies on sophisticated mechanisms involving mediators at molecular and cellular levels. Recent literature has shown that different subcellular vesicles display potent regulatory functions, mediated both by their surface receptors and their content [1]. Among these elements, the biological role of extracellular vesicles (EVs) has been strongly emphasized [2,3].

Extracellular vesicle is the umbrella term for all types of cell-derived vesicles, including microvesicles and exosomes, therefore representing a heterogeneous population of small vesicles deriving from virtually all cell types (i.e., endothelial cells, platelets, leukocytes), and released during cell growth, proliferation, activation, apoptosis, or senescence processes [2,3]. EVs are constantly present in the bloodstream and they have been implicated in the regulating functions of remote organs and tissues [2,3,4,5]. EVs are also characterized by an integral plasma membrane, expressing the phenotype of the cells from which they originate. It has been demonstrated that EVs retain a broad enzymatic repertoire, being able to maintain a number of biological activities even after the budding from their parental cells [6,7,8,9]. Moreover, EVs may represent the indices of cell activation and/or tissue degeneration, occurring during pathophysiological events in vivo.

It has been reported that EVs play a crucial role in a multitude of pathologies, including malignancies, cardiovascular, inflammatory, metabolic, and autoimmune diseases [3,5,10,11,12,13,14]. As a consequence, circulating EVs have been proposed as reliable biomarkers, able to provide relevant information on pathogenic events and response to treatments in several clinical settings [3,15]. However, due to their small size, state-of-the-art protocols for EVs detection requires a number of pre-analytical enrichment steps, such as centrifugation/ultracentrifugation, size exclusion chromatography, ultrafiltration, immunocapture, hydrostatic or hydrostatic filtration dialysis; therefore, their final characterization relies on material that differs from the original body fluid [16,17,18].

In this context, evaluating to which extent the final measurement reflects the initial characteristics of the samples, and how these features have been influenced by pre-analytical processing enrichments may result difficult [18,19,20,21].

Polychromatic flow cytometry (PFC) tends to emerge as a promising technique for EV characterization and enumeration. Due to its sensitivity, flexibility and ability to quickly analyze thousands of events and multiple parameters at the same time, it allows to simultaneously characterize and quantify EVs stemming from different parental cells [17]. Even if typical applications of flow cytometry rely on forward scatter (FSC)/side scatter (SSC) measurements, some data suggest that flow cytometry approaches used to identify EVs, based solely on their scatter parameter detection, underestimate EVs counts [22,23]. It has been also demonstrated that the careful choice of an EVs probe and of the staining conditions allows the application of a fluorescence triggering, which could have a profound impact on the amelioration of the sensitivity of EVs flow cytometry analyses [23,24].

Therefore, the application of a simplified protocol, combined with the possibility of applying a fluorescence triggering, would represent a substantial step forward in the process of unequivocally identifying and enumerating EVs by PFC.

In the present study, we developed a straightforward procedure, which takes advantage of a lipophilic cationic dye (LCD), for the identification, enumeration, and separation of EVs from different origins (platelets, leukocytes or endothelial cells). The protocol was applied on fresh peripheral blood (PB) samples and the LCD staining of EVs allowed the use of a PFC analysis based on the application of a trigger threshold. We show that LCD identifies biological elements that display typical EVs features. Moreover, the application of this newly optimized PFC protocol allows reproducible EVs counts as well as the efficient purification of EVs by fluorescence-activated cell sorting. As demonstrated by proteomics analysis, sorted EVs preparations also carry a protein cargo linked to EVs specific bioactions (i.e., binding processes).

Altogether, these results show that this newly optimized method represents an essential step towards for the study of EVs clinical significance in different pathological settings.

## 2. Results

### 2.1. Flow Cytometry Identification and Subtyping of EVs from Human Whole Peripheral Blood Samples

**Figure 1:**
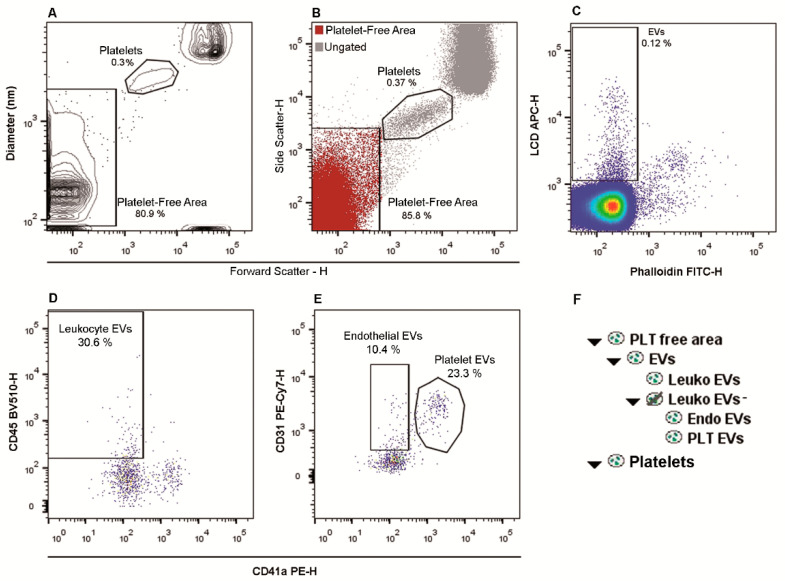
Gating Strategy for extracellular vesicles (EVs) identification and subtyping. (**A**). A platelet-free area region was defined on a Forward Scatter-H/Diameter dot-plot (.fcs files were transformed by the Rosetta system). (**B**). All events were also represented on a Forward Scatter-H/Side Scatter-H dot-plot and, by using platelets as a reference population, the same area free from platelets, evidenced in A, can be gated. (**C**). The “Platelet-free area” was shown on a Phalloidin-H/Lipophilic Cationic Dye (LCD)-H dot-plot and EVs were identified as LCD positive/phalloidin negative events. (**D**). EVs (LCD+/Phalloidin- events) were analyzed on a CD45-H/CD41a-H dot-plot and CD45+ events were identified as leukocyte-derived EVs (Leukocyte EVs). (**E**). A logical gate excluding all the CD45+ events was then obtained, and the resulting population was plotted on a CD31-H/CD41a-H dot-plot. Events showing the CD31+/CD41a+ phenotype were identified as platelet-derived EVs (Platelet EVs), whereas the CD31+/CD41a- compartment represented endothelium-derived EVs (Endothelial EVs). The percentages reported in the dot-plots were calculated based on the gating hierarchy that we have used. (**F**). The applied gating hierarchy is shown as a scheme.

Figure 1A shows a dot-plot, representing the size (in nm) and the FSC-H, used to establish a region, defined as “platelet free area”, under the one in which platelets fall. As shown in Figure 1B, the “platelet free area” matches with a similar area displayed on a related FSC-high (H)/SSC-H dot-plot (Figure 1B). The events of the “platelet free area” were then represented on an LCD-H/Phalloidin-H dot-plot and EVs were identified as LCD positive/phalloidin negative dots (Figure 1C). We focused our attention on the evaluation of CD45+ EVs, which are reported to derive from leukocytes [18,25], as well as on CD41a+/CD31+ EVs, known to stem from platelets [12,18], and on CD41a-/CD45-/CD31+ EVs that have been demonstrated to derive from the endothelial cell compartment [12,18]. Those EVs subtypes are the most widely studied and represented in the PB [18,26]. Therefore, EVs (LCD+/Phalloidin- events) were analyzed on a CD45-H/CD41a-H dot-plot and CD45+ events were identified as leukocyte-derived EVs (Leukocyte EVs, Figure 1D and Supplementary Figure S1F). A CD45 negative logical gate was set and the resulting population was plotted on a CD31-H/CD41a-H dot-plot (Figure 1E and Supplementary Figure S1G). Events showing the CD31+/CD41a+ phenotype were identified as platelet-derived EVs (Platelet EVs), whereas the CD31+/CD41a- compartment were identified as endothelium-derived EVs (Endothelial EVs). In Figure 1F the used gating hierarchy is shown as a scheme.

Supplementary Figure S2 shows reagent-only and buffer-only controls, paralleled to the respective stained sample. The setting of the allophycocyanin (APC) channel trigger threshold produced the acquisition of almost no events during the time interval needed for the sample acquisition (~1 min) when the buffer-only control was acquired. Furthermore, to verify that LCD staining targets intact EVs, three samples were acquired by flow cytometry, treated by a solution of 1% Triton X-100 and then re-acquired. As shown in Figure S1D,E, after the membrane disruption (Triton X-100 treatment), the whole EVs population (LCD+/Phalloidin- events) disappeared. Supplementary Figure S1H–L show fluorescence minus one (FMO) controls (where the respective isotype control was added) for CD45, CD41a, and CD31.

In order to ascertain that the protocol here described did not produce any platelet activation, which could create artefacts in the EVs detection, we have stained three different PB samples with a well-known marker of platelet activation (CD62P, also known as P-Selectin). As shown in Supplementary Figure S3, only a small subset of platelets (PLTs, *n* = 3; mean = 2.66% ± 1.27%) resulted activated (CD62P+) following the application of the method here described.

### 2.2. Sensitivity of the Flow Cytometry Fluorescence Triggering

In order to evaluate the sensitivity of the allophycocyanin (APC)-triggering approach that we described, the fluorescence intensity of a mixture of equivalent reference fluorophores (ERF) calibration particles was measured by SSC triggering, as recommended, and its calibration curve, relating the number of fluorophores of the particles to their fluorescence intensity, was obtained (Supplementary Figure S4B). The ERF calibration allowed us to establish that, by using the above reported threshold on the APC channel, the minimum APC fluorescence value detectable in such a setting resulted 696 ERF. We also acquired LCD-stained liposome samples measuring 103 ± 2.4 nm in diameter observing that, for them, an ERF of 6959 was measured in the APC channel. Therefore, based on the relationship between EVs diameters and LCD fluorescence intensities of liposomes, we could hypothesize that, by applying this method on our instrument, EVs smaller than 100 nm could be detected.

Interestingly, as now shown in Supplementary Figure S5B, we have also analyzed, on three different samples, the expression of CD41a, which resulted higher in platelets than in platelet derived EVs.

### 2.3. Size and Morphology of LCD Positive Events

**Figure 2:**
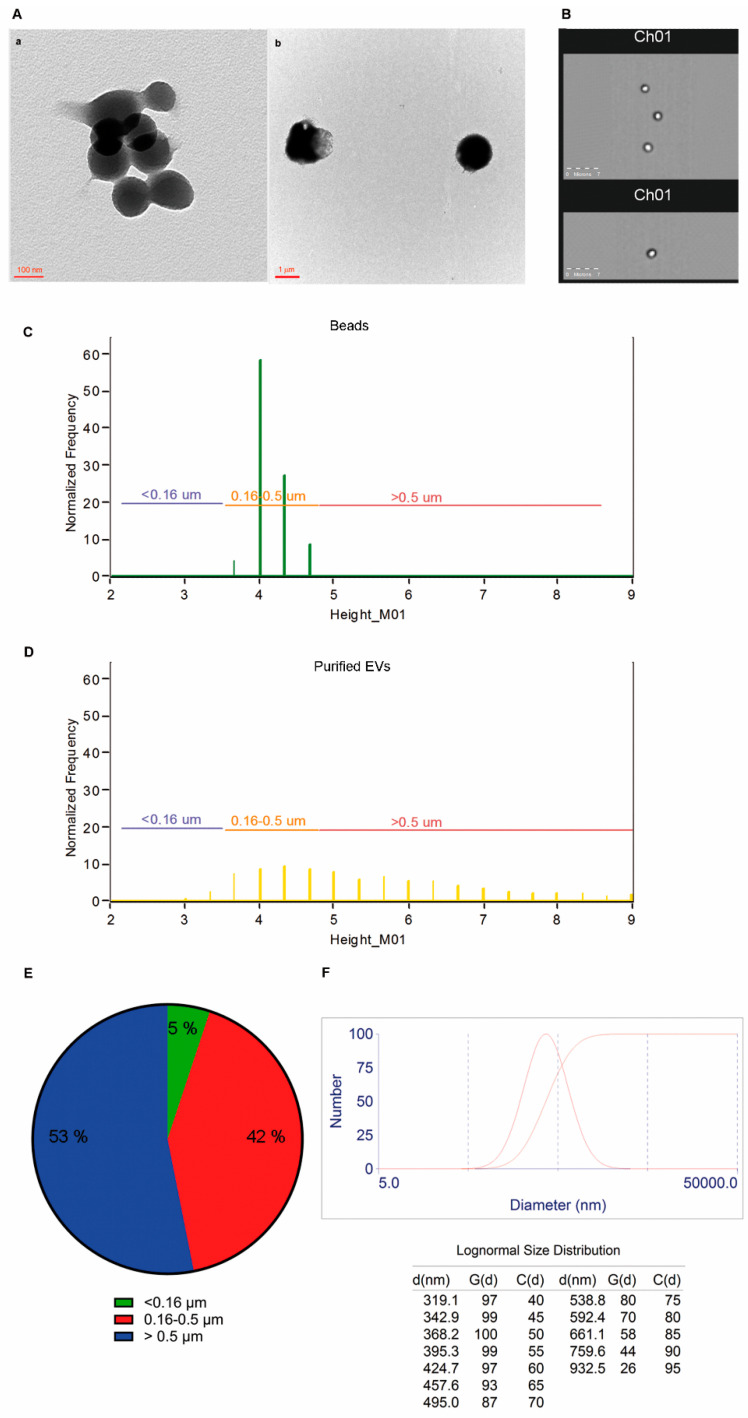
LCD+/Phalloidin- EVs features. (**A**). Transmission electron microscopy micrographs show EVs purified from whole blood samples (scale bar a. 100 nm; b. 1 μm). (**B**). Sorted EVs preparations were analyzed by an ImageStream X Mk II Imaging Flow Cytometer equipped with a 60X lens and some captured brightfield images (Channel 1) were shown. (**C**). MegaMix-Plus SSC beads were acquired by ImageStream X Mk II and shown as a graph representing their diameters (calculated as height signals of the brightfield channel–M01). Three gates were established on this graph: a region encompassing all the events smaller than 160 nm (<0.16 µm), a gate including all the events displaying diameters in the range 160–500 nm (0.16–0.5 µm), and a region enclosing the events bigger than 500 nm (>0.5 µm). (**D**). These three gates were applied to the analysis of the LCD+/Phalloidin- EVs samples, previously separated by activated-cell sorting and then acquired by ImageStream X Mk II, using the same settings used for the acquisition of the beads. (**E**). Percentages of the different EVs diameters were reported as a pie chart (*n* = 3). (**F**). Sorted EVs preparations were analyzed by dynamic light scattering (DLS). G(d) refers to the relevant distribution information data and C(d) to the relevant cumulants data related to the measured autocorrelation function. Data are representative of at least three separate experiments.

LCD+/Phalloidin- events were separated by fluorescence-activated cell sorting. Size and morphological features of sorted EVs preparation are shown in Figure 2. Images obtained by transmission electron microscopy (TEM) analysis showed that sorted EVs preparations of LCD+/Phalloidin negative events display distributions in the range of ~100–300 nm (Figure 2A(a)) or bigger (~900 nm–1 μm, Figure 2A(b)). Therefore, sorted EVs preparations were analyzed by ImageStream X MK II and representative brightfield images are shown in Figure 2B. We also used the different population of MegaMix-Plus SSC beads to define, on the brightfield channel (Channel 1), reference ranges of dimensions (Figure 2C,D).

Given that the brightfield images derive from the transmitted light of the analyzed particles, they are not affected by the problems related to the refraction index differences between plastic beads and biological vesicles. In this way, as shown in Figure 2E, we could observe the dimension distribution of the EVs that we detected by the here presented protocol on a large population of events (5.5 × 10^3^ EVs), and results showed that the majority of the detected particles (>90%) were larger than 160 nm, but also EVs smaller than 160 nm were separated (and therefore detected) by the method here presented. The representative particle size distribution graph, obtained by DLS analyses and represented by a lognormal distribution, showed that sorted EVs preparations have a size distribution in the range of EVs size (Figure 2F).

Altogether, these data demonstrated that LCD+/Phalloidin- events have a size range within the one reported for EVs dimensions.

### 2.4. LCD Stains EVs through A Dual Action Mechanism

LCD stains the whole population of membrane liposomes (Supplementary Figure S6A). Moreover, we hypothesized that LCD may also stain EVs, given that EVs possibly retain the trans-membrane potential. To test this hypothesis, we treated four PB samples with carbonyl cyanide 3-chlorophenylhydrazone (CCCP), an ionophore used as described in the method section. Supplementary Figure S6B shows that LCD fluorescence intensity, measured on CCCP-treated EVs, significantly decreased when compared to the respective controls treated with the CCCP vehicle (DMSO, *p* < 0.001).

### 2.5. The Specificity of LCD Staining

**Figure 3:**
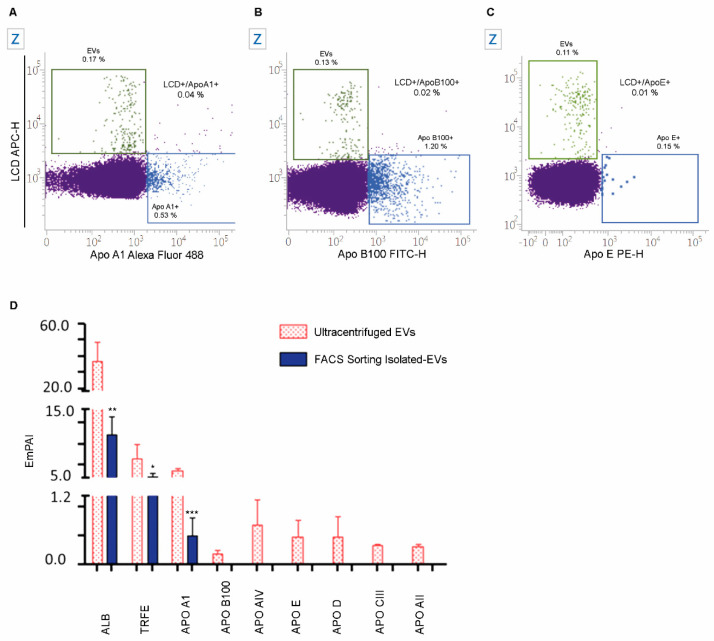
Analysis of the EVs contaminants. Dot-plots represent the parallel analysis of LCD and Apolipoprotein A1 (ApoA1, (**A**)) or Apolipoprotein B100 (ApoB100, (**B**)) or Apolipoprotein E (ApoE, (**C**)) on the population of events resulting phalloidin negative in the “Platelet free area” gate. (**D**). Bars represent the emPAI comparison of known EVs contaminants between sorted EVs (blue bars) and EVs separated by ultracentrifugation (pink bars). Student’s *t*-test, * *p* ≤ 0.05, ** *p* ≤ 0.01, *** *p* ≤ 0.001. Data are representative of three separate experiments.

Given that it has been reported that EVs preparations might be contaminated by low-density lipoproteins (LDL), high-density lipoproteins (HDL), and chylomicrons, and because aggregates of those particles may have the same size of EVs, we have simultaneously stained EVs (with LCD) and ApoB100/B48, ApoA1 or ApoE [16,27]. As shown in Figure 3A–C, few LCD+ EVs are also stained by the used anti-apolipoprotein antibodies (0.01–0.04%). Moreover, a direct comparison of proteins analyzed from EVs purified by fluorescence activated cell sorting (1 × 10^6^ LCD+ Phalloidin- EVs) and the same blood volume used to separate EVs by FACS that instead underwent to EVs enrichment by a classical ultracentrifugation (UC) method [28]. The resulting exponentially modified protein abundance index (EmPAI) values [29] were calculated for the most abundant serum proteins. In detail, Albumin (ALB), Serotransferrin (TRFE), and Apolipoprotein A-I (APOA1), Apolipoprotein B-100 (APOB100), Apolipoprotein AIV (APOAIV), Apolipoprotein E (APOE), Apolipoprotein D (APOD), Apolipoprotein C-III (APOCIII), and Apolipoprotein A-II (APOAII) were evaluated and compared between the two above reported purification conditions. As shown in Figure 3D, with respect to the UC enrichment protocol, the here presented sorting method significantly reduces the serum co-isolated soluble proteins such as ALB (*p* < 0.01), TRFE (*p* < 0.05), and APOA1(*p* < 0.001). Moreover, Figure 3D shows that APOB100, APOAIV, APOE, APOD, APOCIII and APOAII were not detectable in sorted EVs preparations, while they were widely quantified in EVs enriched by UC.

### 2.6. EVs Numbers Obtained by Applying the LCD-Based or the Platelet-Free-Plasma (PFP) Protocol

EVs from three healthy donors were enumerated, in parallel, by the method here described and by the Society of Thrombosis and Haemostasis-promulgated protocol reported for flow cytometry platelet-derived EVs counting. The latter is based on the obtainment of the platelet-free plasma (PFP) fraction, through two consecutive centrifugation steps (2500 g, 15 min, RT) [30,31,32]. Results showed that the concentrations of CD41a+/CD31+ EVs obtained by the two abovementioned protocols resulted in overlapping (whole blood method = 1508.27 ± 957.04 PLT_EVs/μL; standard method = 2584.74 ± 1908.57 PLT_EVs/μL; *p* > 0.05, not significant) [33].

### 2.7. Numbers and Cargo of EVs Isolated from PB Samples of Healthy Volunteers

The application of the newly optimized PFC protocol here described allowed us to obtain highly reproducible EVs counts (CV~3–7%), when EVs concentrations were analyzed in triplicate (3 different tubes) from the PB of the same healthy donor Supplementary Table S1.

**Figure 4:**
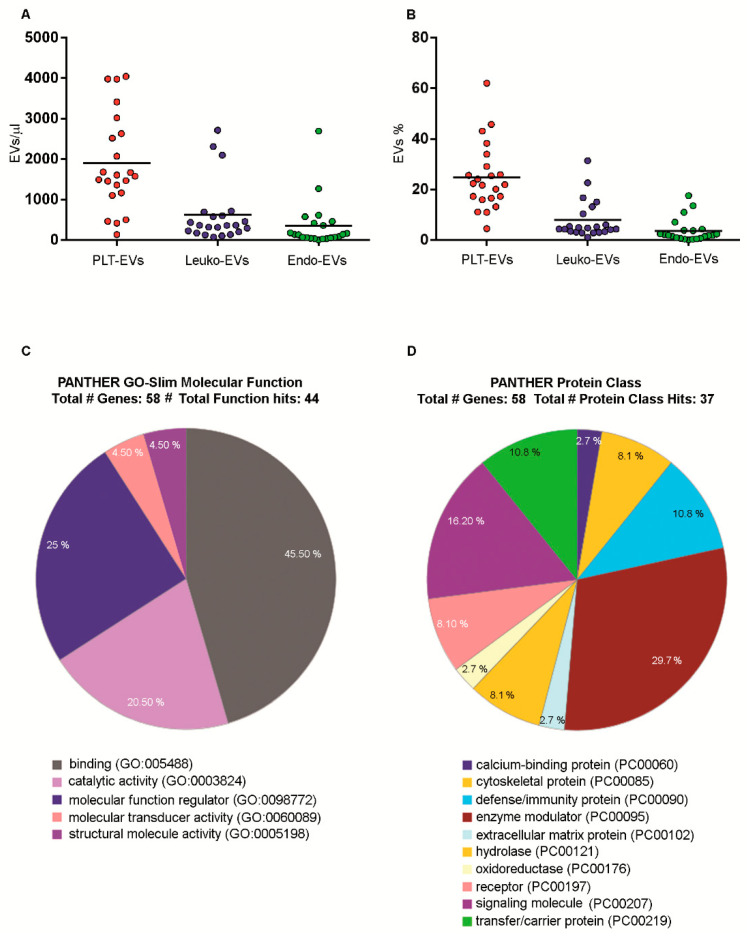
Extracellular vesicle concentrations and percentages. (**A**). The scatter plots represent absolute counts of Platelet-, Leukocyte- and Endothelial-derived EVs. Values are expressed as events/μl. Horizontal lines represent related mean values. (**B**). Scatter plots represent percentages of Platelet-, Leukocyte- and Endothelial-derived EVs. Values are expressed as percentages of total EVs. Horizontal lines represent related mean values. (**C**,**D**). Proteins from sorted EVs preparations were identified by LC MS/MS, then analyzed using PANTHER analysis and finally classified by the Molecular function (**C**) and the Protein class (**D**).

Numbers of EVs and platelet (PLT)-, Leukocyte (Leuko)- and endothelial-derived subsets were then obtained from PB samples of 22 healthy individuals. As shown in Figure 4, resulting EVs concentrations (Figure 4A, Table 1) and percentages (Figure 4B, Table 2) indicated that, among the here detected phenotypes, the most abundant circulating EVs population stems from platelets (median = 1590.90; mean = 1898.31 ± 1182.74 events/μL). Leukocyte- (median = 361.20; mean = 621.06 ± 742.61 events/μL) and endothelial-derived EVs (median = 136.50; mean = 346.63 ± 601.56 events/μL) were also detectable.

In order to understand the characteristics of the cargo conveyed by LCD+/Phalloidin- EVs, they were analyzed by proteomics, after they had been purified by fluorescence-activated cell sorter. The molecular function and the protein class of the identified proteins were reported in Figure 4C,D, respectively, indicating a specific protein organization in the EVs cargos. Results demonstrated that the proteins conveyed by healthy EVs, according to gene ontology analyses of their molecular functions, were classified as having binding functions (GO: 0005488; 45.50%) (Figure 4C). Of note, healthy EVs also convey proteins with regulatory functions (GO: 0098772; 25%), as well as catalytic activity (GO: 0003824; 20.50%). As shown in Figure 4D, by analyzing their protein class, most of them were classified as enzyme modulators (PC: 00095; 29.70%) and signaling molecules (PC: 00207; 16.20%).

## 3. Discussion

EVs are circulating vesicles, generated as a cellular response to different stimuli that contribute to coagulation, inflammation, cellular homeostasis, survival, and waste material transport. EVs have also been shown to be effectors capable of delivering biological messages (mRNA, miRNA, proteins, surface molecules) to target cells [2,3,34,35]. Their concentration, biochemical composition, and cellular origin may give relevant clinical information, and increased numbers of circulating EVs have been observed in a variety of diseases.

Identification and characterization of different types of EVs is challenging due to the lack of appropriate isolation and purification methods. Currently, differential ultracentrifugation, immunoaffinity capture, size-exclusion chromatography and microfluidics are the techniques recommended by the International Society for Extracellular Vesicles (ISEV) [18]. Current protocols, however, require several pre-analytical blood manipulations, that may promote EVs release and/or may induce cell damage affecting the final measurement. For these reasons, the translation of EVs basic research into clinical practice is challenging.

Polychromatic flow cytometry (PFC) represents the method of choice for EVs identification and enumeration. However, the potential for EVs as biomarkers has been limited by the fact that even if a great number of PFC protocols were proposed to identify and enumerate EVs, results from previous studies showed a high degree of variability [31,36].

Nevertheless, when a PFC method for EVs detection in whole blood is optimized, several other considerations about pre-analytical as well as analytical phases must be considered [18,19,20,21]. Taking into account recent methodological guidelines for EVs studies [16,18,32], we demonstrated that the flow cytometry analysis of EVs must be performed on freshly drawn whole blood samples (within 4 h from bleeding), confirming that prolonged storages generate artifacts [32,37,38].

Concerning the flow cytometry analytical phase, we recommend referring to all established guidelines for setting a general PFC panel (i.e., use of the FMO controls, the appropriate gating strategy, the quality controls) [39]. MISEV guidelines for analytical variables, as well as MIFlowCyt and MIFlowCyt-EV suggestions for general variables and experimental design related to flow cytometry EVs experiments were taken into account [18,40,41].

Here we developed a simple procedure, which takes advantage of a lipophilic cationic dye (LCD) for the identification and separation of EVs. It must be underlined that the application of such a flow cytometry method allowed us to detect and enumerate EVs on freshly drawn whole blood samples, avoiding possible artifacts generated by the enrichment procedures (i.e., centrifugation/separation) that can activate or damage cells and artificially generate or disrupt EVs or induce their fusion [32,37,38,42,43,44]. Therefore, the application of EVs analysis on fresh samples reduces the EVs loss problems, preserving the EVs characteristics [45,46].

The here presented approach takes advantage from PFC and it is based on a fast (an hour from bleeding to acquisition) protocol that needs a small volume of whole blood allowing to discriminate intact from damaged EVs in an extensible way.

Furthermore, given that LCD stains the EVs compartment, we could apply the trigger threshold on the channel in which LCD emits, instead of using the triggering on SSC. We then demonstrated that, by this approach, it was possible to detect EVs. Finally, the use of the Rosetta Calibration allowed us to identify the EVs compartment based on size values, therefore distinguishing EVs from other blood elements. As matter of fact, by applying this newly optimized PFC protocol, which also combines the LCD staining to phalloidin for the identification of damaged membranes [47,48], a high level of repeatability, in terms of EVs counts (CV~3–7%) was achieved [23,49,50].

Circulating EVs (endothelium, platelet- and leukocyte-derived EVs) surrounded by an intact plasma membrane were also sub-typed, after optimizing an appropriate panel of antibodies. Data showed that the most abundant population among those that we detected was the one stemming from platelets, followed by the leukocyte-derived population and the one derived from endothelium [12,26,51].

For the first time, by using such a method we were also able to isolate LCD+/Phalloidin- intact EVs by fluorescence-activated cell sorting.

The staining of EVs by LCD also resulted highly specific, given that it excludes HDL, LDL and chylomicron apolipoproteins, which are referred to be the most common EVs contaminates [16,27]. Because the majority of plasma lipoprotein particles have a very small size, these results were confirmed by proteomic that appears as a more suitable method for this type of comparison. Fluorescence activated cell sorting, in fact, giving the possibility to obtain highly purified material, does not allow in any case to separate large numbers of EVs, compatible with some types of analyses (i.e., western blotting). Proteomics data showed that sorted preparations of EVs resulted significantly less contaminated from the soluble circulating components, such as abundant proteins and apolipoproteins, usually co-purified in UC protocols [16,52]. Moreover, by applying this method we obtained similar results when some other biofluids (i.e., cerebrospinal fluid, tears) were analyzed [53]. Furthermore, already published data confirm the nature of LCD+/Phalloidin- EVs, given that, as we have already demonstrated by proteomics, they carry a number of top proteins that were identified in EVs elsewhere [54].

Therefore, these data demonstrated that, the application of the here presented method, allowed the separation of highly purified material from a low volume of samples, particularly suitable for further analyses and potentially useful for EVs detection in many other biofluids [53]. As shown in the pie chart in Figure 4C, which identifies the EVs protein cargo, these EVs resulted functionally organized, carrying binding proteins (possibly associated to their ability to interact with specific target cells), as well as catalytic and regulatory proteins (probably able to modulate a range of functions in their target cells). The identified EVs protein cargo resulted well organized even from a structural point of view, given that the 8.1 % of the identified proteins belong to the cytoskeleton, and are involved in the structure maintenance (Figure 4D). Moreover, 10.8 % of those proteins resulted involved in the immune system-mediated activities and the remaining are functional proteins (enzyme modulators, oxidoreductase, receptors and signaling molecules). These data confirm, for EVs circulating in the blood, some other recently published results obtained for cerebrospinal fluid and tear EVs, indicating a general functional organization of the EVs cargo [53].

## 4. Materials and Methods

### 4.1. Samples

The study was approved by the local ethics committee of Chieti-Pescara and University G. d’Annunzio, Chieti-Pescara (V. 1.0, 4 February 2016). All participants and activities were conformed to the current legislation and regulations in Italy, European Legislation, International Conventions and Declarations. In accordance with the Helsinki II Declaration, all involved subjects gave written informed consent before their inclusion in the study, and participants were identified by anonymized codes. Peripheral blood (PB) samples were obtained from 53 healthy Caucasian donors that gave written informed consent. They did not declare to be under chronic therapies, nor to be affected by chronic conditions. Samples from 31 healthy donors were used for protocol optimization, while the remaining 22 healthy volunteers, whose demographic characteristics are reported in Supplementary Table S2, were analyzed for the assessment of EVs concentrations and phenotypes.

### 4.2. Flow Cytometry EVs Staining

Collection and staining of PB samples. PB was drawn (21 G needles) in two sodium citrate tubes (Becton Dickinson Biosciences-BD, San Jose, CA, USA, Ref 454387) and processed within 4 h from venepuncture. The first harvested tube was discarded to minimize venepuncture-induced vascular damage effects [55]. To obtain a method for a rigorous EVs definition, different known EVs tracers were tested (Supplementary Table S3). Among them, LCD resulted in the most promising marker, giving the best separation of the positive population with respect to the related internal negative one (Supplementary Figure S7). The best combination of markers for EVs analysis was obtained after testing different reagent combinations (Supplementary Table S4). In order to stain PB samples, a reagent mix was prepared by adding to 195 μL of PBS 1×, 0.5 μL of Fluorescein isothiocyanate (FITC)-conjugated phalloidin and LCD, and all reagents, as detailed in Supplementary Table S5; then 5 μL of whole blood were added to the mix. The lipophilic cationic dye is a commercial compound, that we have validated and patented for its off label use to stain EVs for further flow cytometry analysis. The chemical structure of this molecule is not public. BD Biosciences produces the custom LCD kit on the basis of customer requests. Given that LCD kit is a custom product, it is not reported on standard catalogues, but related reference numbers are 626266 (antibodies, listed in Supplementary Table S5) and 626267 (LCD and FITC-conjugated phalloidin, Supplementary Table S5). To avoid immune complex formation and the unspecific background linked to the antibody aggregation, each reagent stock solution was centrifuged before its use (21,000× *g*, 12 min). After 45 min of staining (RT, in the dark, or at 37 °C when Annexin V was not present in the reagent mix), 500 μL of PBS 1× were added to each tube and 1 × 10^6^ events/sample were acquired by flow cytometry (FACSVerse, BD Biosciences, San Jose, CA, USA). In a subset of samples, Peridinin Chlorophyll Protein-Cyanin (PerCP-Cy) 5.5-conjugated Annexin V was also added (0.25 µL, BD Biosciences, Cat: 561431), and, in this case, Binding Buffer 1X (BD Biosciences) was used instead of PBS 1X. The dilution of the sample was optimized, and, at the used dilution (1:143), no swarm effects occur (Supplementary Figure S5A) [56].

All requirements imposed for polychromatic flow cytometry EVs analysis were taken into account [18,40,41,48]. In detail, MISEV guidelines for analytical variables, as well as MIFlowCyt and MIFlowCyt-EV suggestions for general variables and experimental design related to FC EVs experiments were taken into account.

#### 4.2.1. Analysis of Platelet Activation

The reagent mix was prepared by adding 3 μL of CD41a PerCP-Cy 5.5-conjugated (BD Biosciences, Cat: 333148), 20 μL CD62P R-phycoerythrin (PE, BD Biosciences, Cat: 555524) and LCD, phalloidin-FITC, CD31 PE-Cyanine 7 (PE-Cy7)-conjugated and CD45 Brilliant Violet 510 (BV510)-conjugated (Supplementary Table S5); then 5 µL of whole blood were added to the mix for 45 min (37 °C, in the dark), then 500 µL of PBS 1X were added to each tube before the acquisition.

#### 4.2.2. Staining of Apolipoproteins

The reagent mix was prepared by adding to 195 µL of PBS 1×, 0.5 μL of LCD and all reagents listed in Supplementary Table S6; then 5 μL of PB were added to the mix and incubated (45 min, 37 °C, in the dark); 500 μL of PBS 1X were added to each tube before the acquisition.

### 4.3. Flow Cytometry Extracellular Vesicle Acquisition and Analysis

The trigger threshold was placed on the channel in which LCD emits (Allophycocyanin—APC—channel, threshold value = 200/262,144). MegaMix-Plus beads (Byocitex, Marseille, France) were measured in order to verify overtime the correct placement of the gating on the scattered dot-plots. For all used parameters the height (H) signals, as well as bi-exponential or logarithmic modes were selected. Rosetta Calibration (Exometry, Amsterdam, The Netherlands) was used, according to manufacturer’s specifications, to calibrate side scatter, relate side scatter in arbitrary units to standardized units of nm, as well as to the diameter and refractive index of particles [57]. Throughout all other measurements, we used the same settings. The EVs/SSC relationship was automatically obtained by Mie theory, considering the optical configuration of the instrument (FACSVerse) and assuming a particle refractive index core of 1.40 (Figure 1A, Supplementary Figure S4A) [36,57].

Instrument performances, data reproducibility, and fluorescence calibrations were sustained by the Cytometer Setup & Tracking Module (BD Biosciences). The evaluation of non-specific fluorescence was obtained by acquiring FMO combined with the respective isotype control [49,58]. To ascertain LCD staining, Triton X-100 1% (*n* = 3), buffer only and reagent only controls were acquired (Supplementary Figures S1D,E and Figure S2) [8]. Compensation was assessed using CompBeads (BD Biosciences) and single stained fluorescent samples. Data were analyzed using FACSuite v 1.0.6.5230 (BD Biosciences) and FlowJo X v 10.0.7 (BD Biosciences) software. EVs concentrations were obtained by the volumetric count function [59,60]. The ERF for FITC, PE and APC channels were calculated using Ultra Rainbow Quantitative Particle Kit (Spherotech, Lake Forest, IL, USA, Cat. Number URQP-38-6K), following the manufacturer’s instructions.

### 4.4. Synthesis and Staining of Rhodamine-Liposomes

Thin layer evaporation and extrusion methods were used to synthesize non-fluorescent liposomes or Rhodamine-DHPE liposomes, as previously reported [23,61,62]. Liposomes extruded by 100 nm membrane filters were measured by DLS (average size = 103 nm) and stained (100 µL) by adding LCD (BD Biosciences) at the same concentration used for EVs detection; then 500 µL of PBS 1X were added to the samples and analyzed by flow cytometry (FACSVerse, BD Biosciences).

### 4.5. Carbonyl Cyanide 3-Chlorophenylhydrazone Impact on LCD Staining of Extracellular Vesicles

To ascertain the possibility that LCD staining is also linked to the EVs trans-membrane potential, four PB samples were initially treated by 50 μM (CCCP; Sigma-Aldrich, Corporation, St. Louis, MO, USA) or its vehicle (0.1% DMSO) for 15 min at RT, and then stained by LCD and phalloidin-FITC, and finally acquired by flow cytometry.

### 4.6. EVs Separation by Fluorescence-Activated Cell Sorting

In order to separate EVs by fluorescence activated cell sorting, PB samples were stained by a reagent mix prepared as above described. Briefly, 0.5 µl of FITC-conjugated phalloidin and LCD (BD Biosciences–Catalogue, #626267, Custom Kit), and all reagents, as detailed in Table S5, were added to 195 µL of PBS 1X; then 5 µL of whole blood were added to the mix. After 45 min of staining (RT, in the dark, or at 37 °C), at least 500 µL of PBS 1X was added to each tube. Such a dilution allowed us to maintain the correct event rate recommended for the nozzle that we have used (100 μm nozzle).

The total EVs fraction and/or PLT-derived CD41a+ EVs (gated as shown in Figure 1) were separated (100 μm nozzle) from PB samples by a fluorescence-activated cell sorter (FACS, FACSAria III, BD Biosciences) [23,48,53,63]. The instrument was set as already described for the analyzer. In detail, the trigger threshold was placed on the APC channel and, for all parameters, the height (H) signals, as well as bi-exponential or logarithmic modes were selected. The post-sorting purity (Supplementary Figure S1A–C) was assessed by using the same instrument (FACSAria III) and the same setting applied for EVs separation [23,48,53]. Instrument performances, data reproducibility, and fluorescence calibrations were sustained by the Cytometer Setup & Tracking Module (BD Biosciences).

### 4.7. Transmission Electron Microscopy Analysis

A Formvar/Carbon 300 Mesh Nickel grid (Agar Scientific, Stansted, UK, Cat: S162N3) was placed on the bottom of a polypropylene tube (14 × 89 mm, Beckman Coulter, Brea, CA, USA, Ref: 331372), filled by the suspension of FACS-purified EVs, and centrifuged (100,000× *g*, 70 min, 4 °C, max brake setting; Optima XL-100K ultracentrifuge, rotor = SW 41 Ti Swinging-Bucket Rotor, Beckman Coulter). Samples were then fixed by using 1% Glutaraldehyde in 0.1 M Cacodylate Buffer (pH 7.4). Images were acquired by a transmission electron microscope ZEISS 109 equipped with a Gatan-Orius SC200W-Model 830.10W TEM CCD Camera.

### 4.8. Dynamic Laser Light Scattering (DLS) Analysis of Purified EVs

Extracellular vesicles, purified as above described by FACS, were analyzed by DLS (90Plus/BI-MAS Zeta Plus, Brookhaven Instrument Corp, Holtsville, NY, USA). EVs size was obtained from the translational diffusion coefficient, by using the Stokes–Einstein equation. DLS data, referring to the intensity of scattering of the samples, were analyzed.

### 4.9. ImageStream Analysis of EVs

MegaMix-Plus beads SSC were used as reference material for dimensions on the brightfield channel. EVs were stained and separated by FACS, as aforementioned. EVs preparations were then immediately acquired on an ImageStream X Mk II (AMNIS Seattle, Seattle, WA, USA) imaging flow cytometer (using a 60 × objective), equipped with the Inspire software (V.200.1.620); data were analyzed by IDEAS software v6.2 (AMNIS). At least 5.5 × 10^3^ sorted EVs/sample were recorded.

### 4.10. EVs Label-Free Proteomics

The number of purified EVs, established by FACS, was used to normalize proteomics analyses. In detail, 1 × 10^6^ EVs separated by FACS were used for each proteomic detection as previously reported [53,63]. EVs digested proteins were acquired in triplicate by LC-MS/MS using a Proxeon EASY-nLCII (Thermo Fisher Scientific, Milan, Italy) chromatographic system coupled to a Maxis HD UHR-TOF (Bruker Daltonics GmbH, Bremen, Germany) mass spectrometer. In-source reference lock mass (1221.9906 m/z) was acquired online throughout runs. Protein identification was carried out by the MASCOT search engine, assuming the carbamidomethylation and the methionine oxidation as fixed and variable modification, respectively. The Exponentially Modified Protein Abundance Index (emPAI) [29] obtainable by MASCOT results from MS/MS data was used for approximate relative abundance of proteins in the mixture. Gene Ontology classification of identified proteins was carried out by PANTHER, which classified 58 identified EVs proteins. The mass spectrometry proteomics data have been deposited to the ProteomeXchange Consortium via the PRIDE [64] partner repository with the dataset identifier PXD022807.

### 4.11. Statistical Analysis.

Flow cytometry and emPAI protein data were analyzed using the XLSTAT 2014 (Addinsoft, Paris, France) and GraphPad Prism 6 (GraphPad Software Inc., La Jolla, CA, USA). Two-sided Student’s *t*-test or paired *t*-test were used as indicated. Statistical significance was accepted for *p*  < 0.05.

## 5. Conclusions

In conclusion, the direct analysis of EVs from freshly drawn whole blood samples, in combination with the staining by LCD (and the possibility to apply a fluorescence trigger), phalloidin and an appropriate antigen panel, represents the major advantage of the protocol described in this study. Such a method, allowing accurate discrimination among EVs and artifacts, is key to study different circulating EVs phenotypes and to assess the predictive/prognostic significance of EVs concentrations in different clinical settings, whenever fresh samples can be obtained. Furthermore, through this method, EVs can be enriched by fluorescence-activated cell sorting, which may give rise to the possibility of analyzing the hypothesized role of EVs as key players of a communication network for the local and the systemic exchange of biological information. These concepts, applied to human studies, could open novel perspectives in the translational medicine field, both in the diagnostics and in the therapy.

## Figures and Tables

**Table 1:** Absolute numbers of EVs and EVs subtypes in healthy PB samples.

*Statistic*	Total EVs/μL	Endo EVs/μL	Leuko EVs/μL	PLT EVs/μL
No. of observations	22.00	22.00	22.00	22.00
Minimum	613.20	0.00	71.40	134.40
Maximum	15,569.40	2695.00	2716.00	4044.60
Median	8997.80	136.50	361.20	1590.90
Mean	8323.57	346.63	621.06	1898.31
Standard deviation (n-1)	4214.35	601.56	742.61	1182.74

**Table 2:** Proportion of EVs subtypes in healthy PB samples.

*Statistic*	% Endo EVs	% Leuko EVs	% PLT EVs
No. of observations	22	22	22
Minimum	0.00	1.1	4.5
Maximum	17.5	31.4	62.0
Median	1.9	4.6	22.1
Mean	3.6	7.8	24.8
Standard deviation (n-1)	4.6	7.6	13.2

## Supplementary Materials

Supplementary materials can be found at https://www.mdpi.com/1422-0067/22/1/48/s1.

## Abbreviations

APC	Allophycocyanin
BV	Brilliant Violet
CCCP	Carbonyl cyanide 3-chlorophenylhydrazone
DMSO	Dimethyl Sulfoxide
DLS	Dynamic Light Scattering
emPAI	Exponentially Modified Protein Abundance Index
ERF	Equivalent reference fluorophores
EVs	Extracellular vesicles
FC	Flow cytometry
FSC	Forward scatter
FITC	Fluorescein isothiocyanate
FMO	Fluorescence Minus One
LCD	Lipophilic Cationic Dye
PB	Peripheral Blood
PBS	Phosphate-buffered saline
PE	Phycoerythrin
PE-Cy7	Phycoerythrin-Cyanine 7
PerCP-Cy5.5	Peridinin Chlorophyll Protein- Cyanine 5.5
PFC	Polychromatic flow cytometry
PFP	Platelet-Free Plasma
SSC	Side scatter
TEM	Transmission Electron Microscopy
UC	Ultracentrifugation
