# Supplementary material for: Flow Cytometry Analysis of Circulating Extracellular Vesicle Subtypes from Fresh Peripheral Blood Samples
Source: Int J Mol Sci. 2020 Dec 23;22(1):48. doi: 10.3390/ijms22010048 (PMC7793062; doi:10.3390/ijms22010048)
Supplement: Supplementary file 1 [file ijms-22-00048-s001.pdf]

Supplementary Figure 1

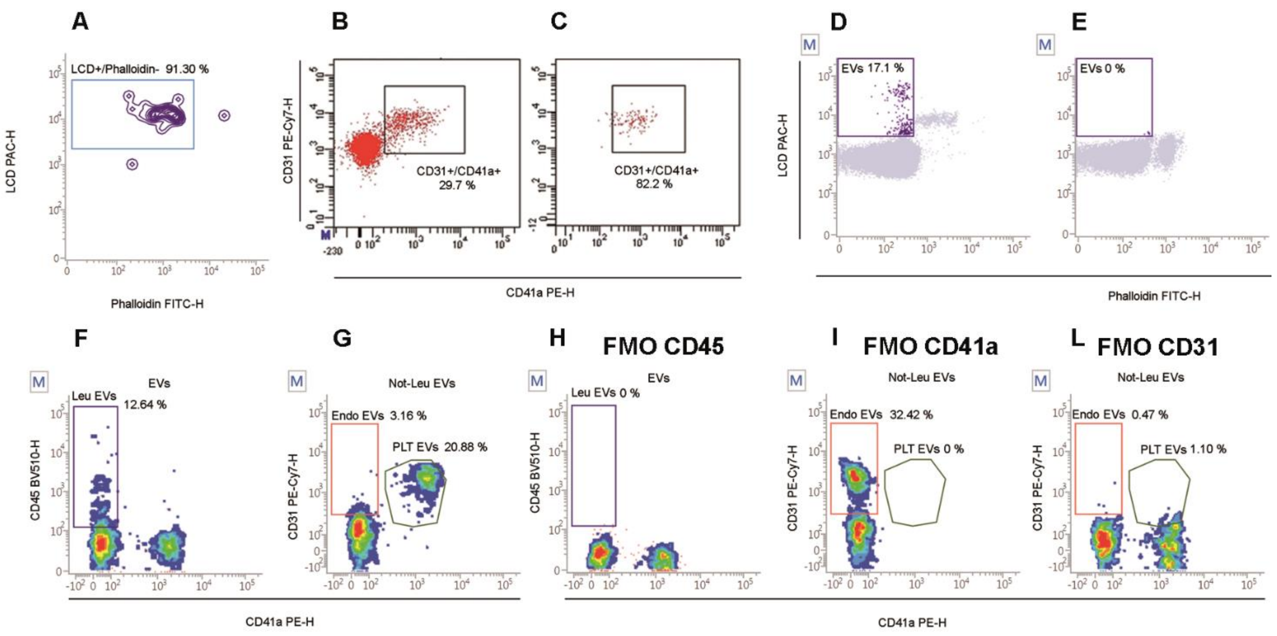

Supplementary Figure 2

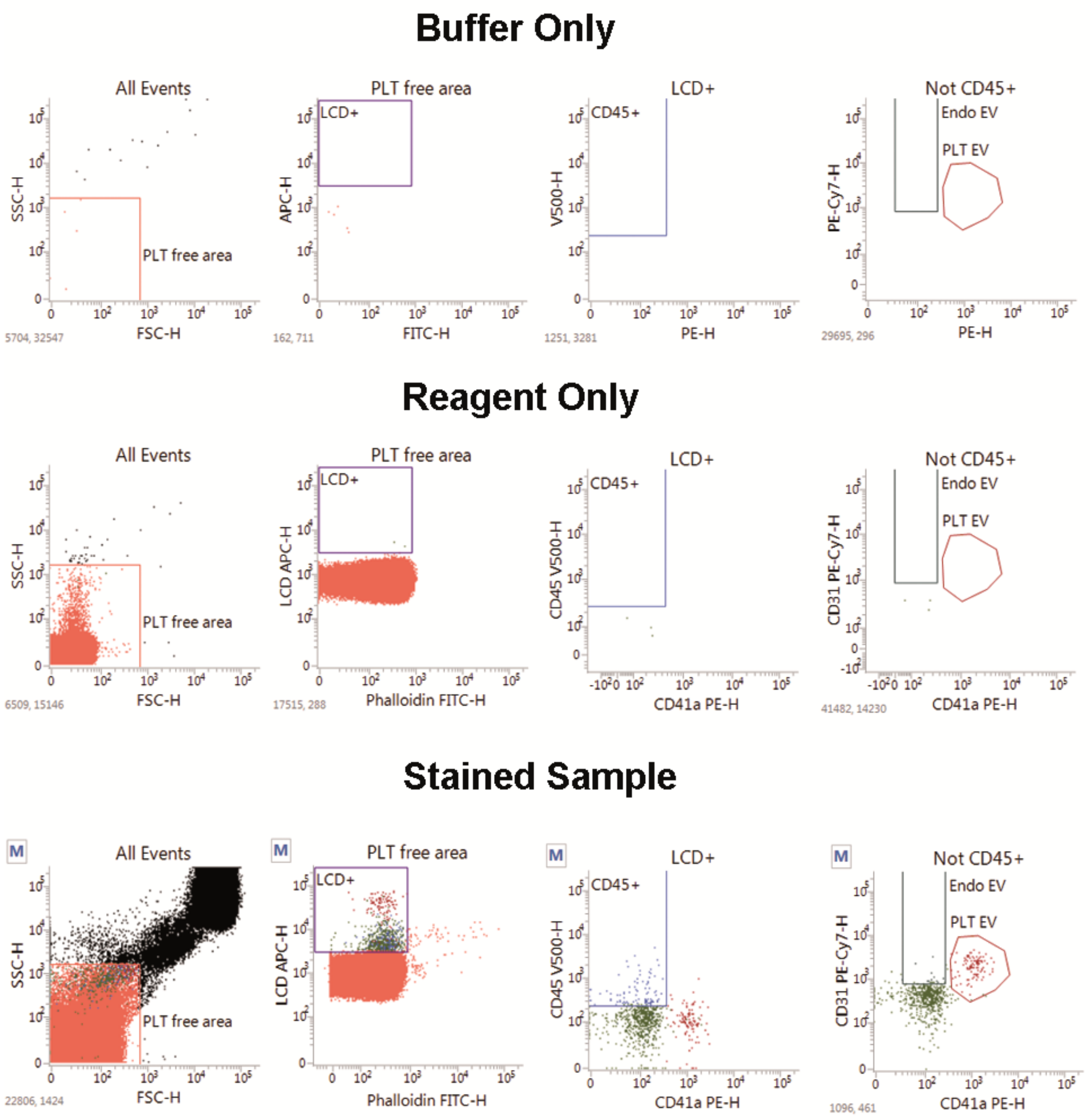

Supplementary Figure 3

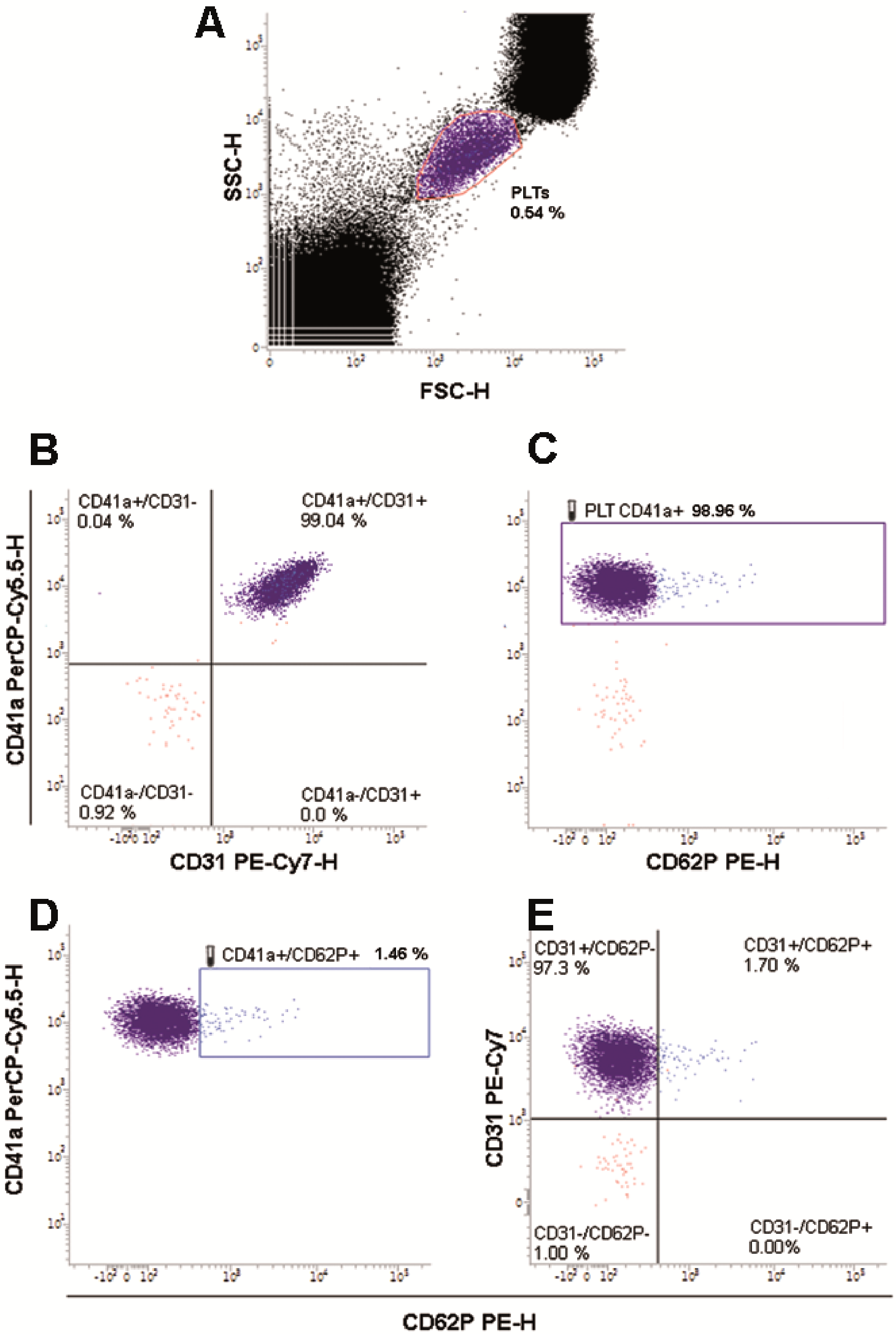

Supplementary Figure 4

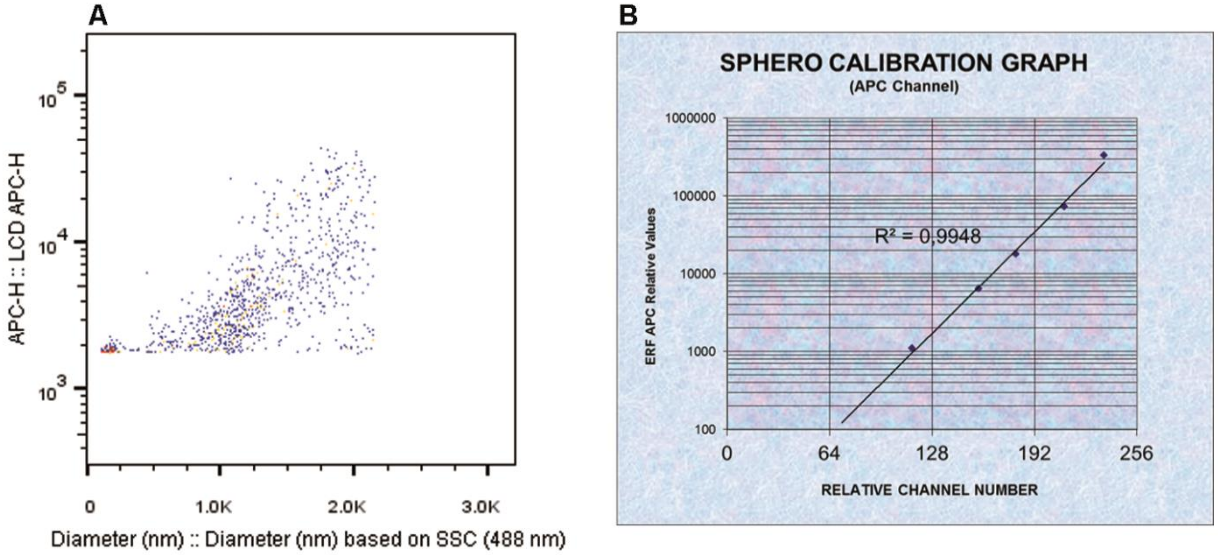

Supplementary Figure 5

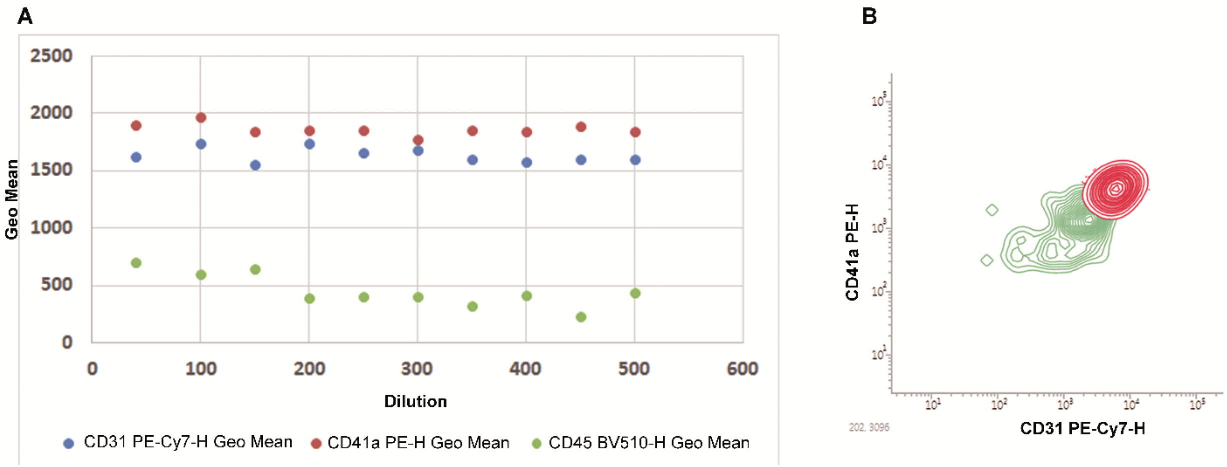

Supplementary Figure 6

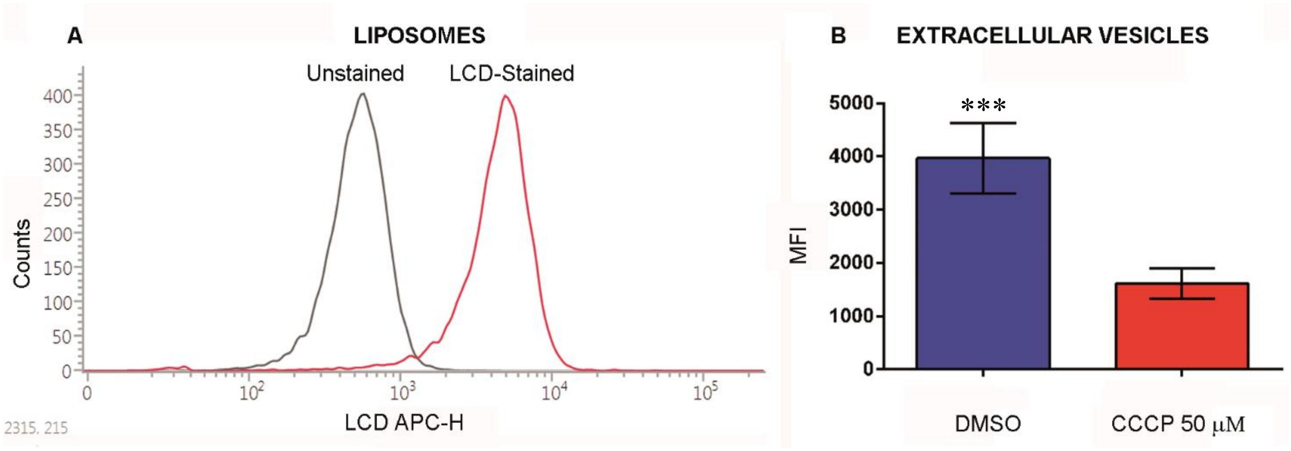

Supplementary Figure 7

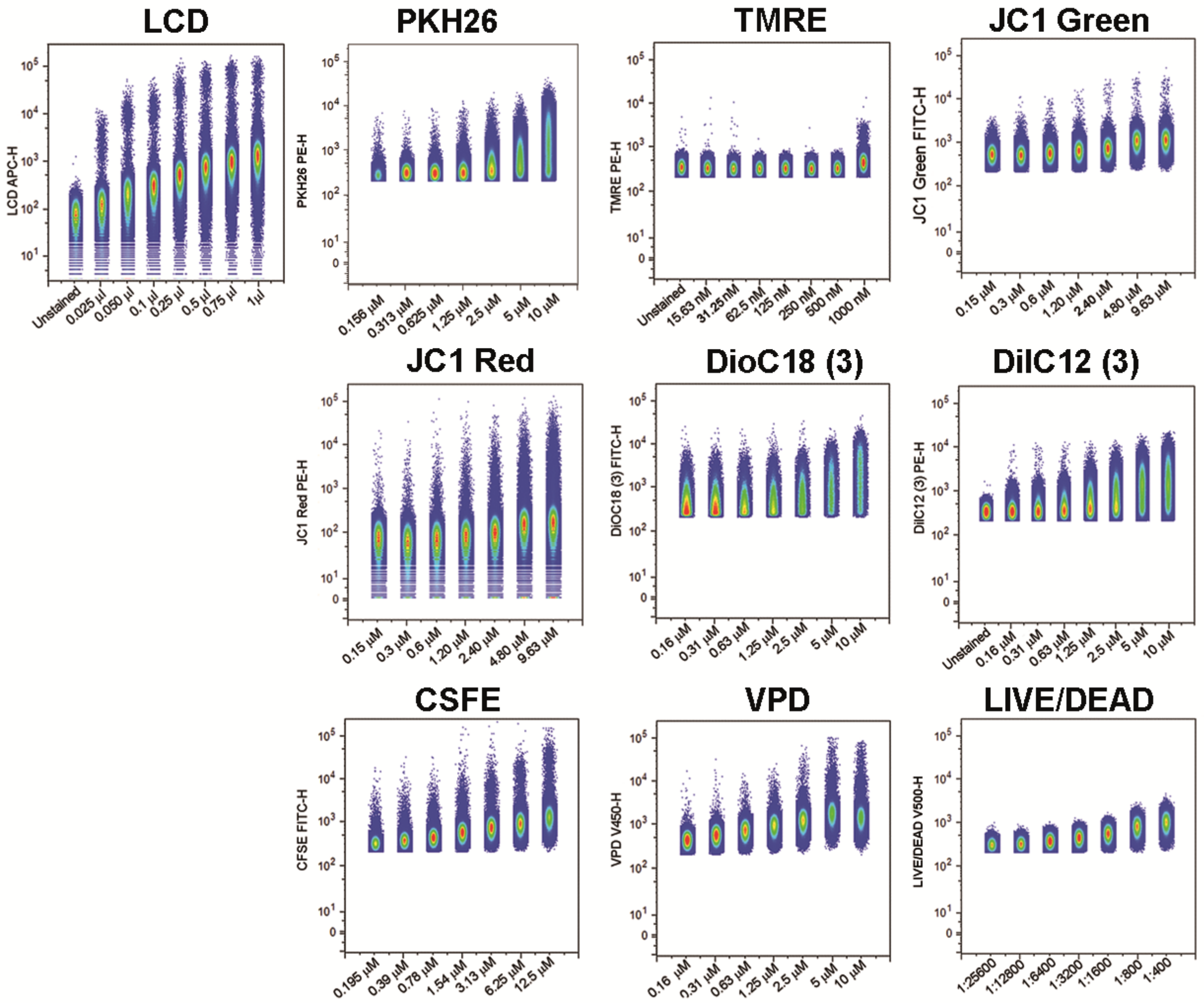

LEGENDS TO SUPPLEMENTARY FIGURES

Supplementary Figure 1. Controls for flow cytometry EV analysis. A. The Phalloidin-FITC/LCD dot-plot shows the purity of extracellular vesicles separated by a FACSAria III (BD Bioscience) fluorescence-activated cell sorter. **B**. EVs (LCD+/Phalloidin-) were gated the platelet-derived compartment was displayed and separated by fluorescence-activated cell sorter (FACSAria III acquisition). **C**. The purity of platelet derived EVs is shown (FACSAria III acquisition). **D.** The dot-plot shows EVs from a whole blood sample stained by LCD and FITC-conjugated Phalloidin (Data were obtained by using a FACSVerse flow cytometer). **E.** The same sample was treated by 1% Triton X-100, and then re-acquired (Data were obtained by using a FACSVerse flow cytometer). **F-G** The dot-plot shows EVs from a whole blood sample identified as described. LCD+/Phalloidin- events were represented on CD45/CD41a dot plot (**F**). CD41a-/CD45-/CD31+ EVs (Endo EVs) are represented on CD31/CD41 dot plot. **H.** Fluorescence minus one control with the respective isotype control for CD45 BV510. **I.** Fluorescence minus one control with the respective isotype control for CD41a PE. **L.** Fluorescence minus one control with the respective isotype control for CD31 PE-Cy7. Data are representative of three separate experiments.

Supplementary Figure 2. Buffer-only and Reagent-only controls.
Buffer-only and Reagent-only controls were acquired and paralleled with a stained sample of peripheral blood.

Supplementary Figure 3. Platelet activation measurement. A. Platelets were identified on a FSC-H/SSC-H dot plot and then they were analysed for their positivity to CD31 and CD41a (on a CD31-H/CD41a-H dot-plot) (**B**). **C.** CD41a+ Platelets were gated. **D.** CD41a+ Platelets were represented on a CD62P-H/CD41a-H and CD62P+ Platelets were identified. **E.** CD41a+ Platelets were represented on a CD62P-H/CD31-H dot-plot. Data are representative of three separated experiments.

Supplementary Figure 4. Fluorescence evaluation and ERF calibration. A. EVs defined as previously described (LCD+/Phalloidin- in the "PLT free area") were represented in a Diameter (nm)/LCD-H dot plot. **B.** The ERF calibration curve for APC relative values obtained by the acquisition of Ultra Rainbow Quantitative Particle Kit is represented.

Supplementary Figure 5. Analysis of marker expression. A. Geo mean values for CD31-PE-Cy7-H (blue dots), CD41a PE-H (red dots), CD45 BV510-H (green dots) of LCD+/Phalloidin- EVs were reported for a number of serial dilutions. **B.** The fluorescent intensities of CD31 and CD41a were measured on Platelets (previously identified on the FSC-H/SSC-H dot-plot) and here represented in red and on platelet-derived EVs, shown in green. Data are representative of three separate experiments.

Supplementary Figure 6. Lipophilic cationic dye staining mechanism. **A**. LCD was used to stain liposomes. The reported histograms represent the LCD stained population (red line) and the overlay of the unstained liposome population (black line). **B**. Blood samples from four different donors were treated by 50 μM CCCP for 15 minutes or by the CCCP vehicle (DMSO). Bars represent LCD MFI values and their respective error bars (± standard deviation) of EVs treated by CCCP or its vehicle (DMSO),Student's *t*-test ***=P≤0.001.

Supplementary Figure 7. Tracers for flow cytometry EV staining. The probes listed in Supplemental Table 2 were tested in human whole PB samples. All these probes were titrated under assay conditions and the related Phalloidin negative population of the "PLT-free area" events were represented for each titration and each probe, as concatenated files.

Supplementary Table 1. Intra-assay Coefficient of variation.			
	Run 1 PLT EVs/µl	***Run 2 PLT EVs/µl***	***Run 3 PLT EVs/µl***
Tube 1	1061.20	1314.60	942.20
Tube 2	1006.60	1283.80	1082.80
Tube 3	1058.40	1176.00	1019.20
Mean	1042.07	1258.13	1014.73
Standard deviation (n-1)	30.75	72.78	70.41
Coefficient of variation	2.95	5.78	6.94
Data related to tubes 1-3 refer to three different tubes of the same sample acquired three time each (run 1-3)			

Supplementary Table 2. Demographic characteristics of healthy volunteers

	Statistic	
	Healthy subjects	22
Age	Minimum	19
	Maximum	52
	Median	34
	Mean	34
	Standard deviation (n-1)	10
Sex	Male	13
	Female	9

Supplementary Table 3. EV Tracers

**Tracers for EV staining**	**Vendor**	**Catalogue Number**
PKH26	Sigma Aldrich	PKH26GL
TMRE	BD Biosciences	564696
JC-1	BD Biosciences	551302
DiOC18(3)	Thermo Fisher Scientific	D275
DiIC12(3)	Thermo Fisher Scientific	D383
CSFE	Thermo Fisher Scientific	C34554
Violet Proliferation Dye VPD450	BD Biosciences	562158
LIVE/DEAD™ Fixable Aqua Dead Cell Stain Kit	Thermo Fisher Scientific	L34957

Supplementary Table 4. Flow cytometry tested reagents

Reagents	Reason of not to be Used
**CD45 BV510**<br>CD45 APC-H7<br>CD45 FITC<br>CD45 APC<br>CD45 V500	FITC: Phalloidin gave the best identification of damaged vesicles;<br>APC: LCD APC was assigned a higher priority;
**CD31 PE-Cy7**<br>CD31 V450<br>CD31 FITC	FITC: Phalloidin was assigned a higher priority<br>V450: CD235a BV421 was assigned higher priority
**CD41a PE**<br>CD41a PerCP-Cy5.5<br>CD41a APC-H7<br>CD41a APC	APC-H7: CD45 was assigned a higher priority.<br>APC: LCD APC was assigned a higher priority
**Annexin V PerCP-Cy5.5**<br>Annexin V APC	APC: LCD APC was assigned a higher priority;
7-AAD	-
CD235a BV421<br>CD235a FITC<br>CD235a PE<br>CD235a APC	CD235a BV421 gave the best identification of erythrocyte derived EVs.
CD34 PE-Cy7<br>CD34 PerCP	Not used: CD34 has lower signal than CD31 for Endothelial EV subpopulation.
CD144 FITC<br>CD144 HV450	Not used: CD144 does not identify any endothelial EV subpopulation.
CD63 PE<br>CD63 FITC	Not used: CD63 is a not very specific marker and it is not highly expressed on the surface of the EVs.
CD309 FITC<br>CD309 PerCPCy5.5<br>CD309 Alexa647<br>CD309 APC	Not used: CD309 does not identify any endothelial EV subpopulation.
CD133 PE<br>CD133 APC	Not used: CD133 does not identify any endothelial EV subpopulation.
CD146 PE<br>CD146 PECy7	Not used: CD146 does not identify any endothelial EV subpopulation.
CD71 FITC	Not used: CD71 does not identify any erythroid EV subpopulation.
CD75 Alexa647	Not used: CD75 does not identify any erythroid EV subpopulation.
Reagents composing the basic panel are evidenced in bold face. Allophycocyanin (APC); APC-Hilite®7 (APC-H7), fluorescein isothiocyanate (FITC), Horizon V450 (V450); Horizon V500 (V500); R-phycoerythrin (PE); PE-Cyanine 7 (Cy7), Peridinin Chlorophyll Protein (PerCP), 7-Aminoactinomycin D (7-AAD), Violet Proliferation Dye 450 (VPD). Bold represents the reagents used.	

Supplementary Table 5. Reagent list

Reagent	*Fluorochrome/Reagent*	*Vendor*	*Clone*	*Catalogue Number*	*Volume per test (μl)*
Lipophilic Cationic Dye (LCD)	-	BD Biosciences	-	626267 custom	0.5
Phalloidin-FITC	FITC	BD Biosciences	-	626267 custom	0.5
CD41a	PE	BD Biosciences	HIP8	626266 custom	5
CD31	PE-Cy7	BD Biosciences	WM59	626266 custom	5
CD45	BV510	BD Biosciences	HI30	626266 custom	5

FITC=Fluorescein isothiocyanate; PE= R-phycoerythrin; PE-Cy7= PE-Cyanine 7, BV=Brilliant Violet.

Supplementary Table 6. Reagent mix for the staining of Apolipoproteins

Reagent	***Fluorochrome/ Reagent***	***Vendor***	***Clone***	***Catalogue Number***	***Volume per test (µl)***
			TUBE 1		
Phalloidin-iFluor 405	iFluor 405	Abcam	-	ab176752	0.2 µl
Anti-ApolipoproteinB-100	FITC	MyBioSource		MBS390004	0.5 µl
			TUBE 2		
Phalloidin-FITC	FITC	BD Biosciences	-	626267 custom	0.5 µl
Anti-Apolipoprotein E	PE	Novus Biologicals	WUE-4	NB110-60531PE	0.5 µl
			TUBE 3		
Phalloidin-iFluor 405	iFluor 405	Abcam	-	ab176752	0.2 µl
Anti-Apolipoprotein A-I	Primary	Santa Cruz Biotechnology		Sc-30089	0.5 µl
Anti-rabbit secondary antibody	Alexa Fluor 488	Thermo Fisher Scientific	-	A11008	1 µl
FITC=Fluorescein isothiocyanate; PE= R-phycoerythrin					
